# Factors associated with early introduction of complementary feeding and consumption of non-recommended foods among Dutch infants: the BeeBOFT study

**DOI:** 10.1186/s12889-019-6722-4

**Published:** 2019-04-08

**Authors:** Lu Wang, Amy van Grieken, Laura A. van der Velde, Eline Vlasblom, Maaike Beltman, Monique P. L’Hoir, Magda M. Boere-Boonekamp, Hein Raat

**Affiliations:** 1 Department of Public Health, Erasmus University Medical Center, PO Box 2040, 3000 CA Rotterdam, the Netherlands; 2 Department of Public Health and Primary Care, Leiden University Medical Center, Den Haag, the Netherlands; 3 TNO Child Health, Leiden, the Netherlands; 4 Department of Agrotechnology and Food Sciences, Subdivision Human Nutrition, Wageningen University and Research, Wageningen, the Netherlands; 5 Department Health Technology and Services Research, Technical Medical Center, University of Twente, Enschede, the Netherlands

**Keywords:** Introduction of complementary feeding, Sweet beverage, Snack foods, Risk factors

## Abstract

**Background:**

Timing and types of complementary feeding in infancy affect nutritional status and health later in life. The present study aimed to investigate the factors associated with early introduction of complementary feeding (i.e., before age 4 months), and factors associated with infants consumption of non-recommended foods, including sweet beverages and snack foods.

**Methods:**

This study used cross-sectional data from the BeeBOFT study (*n* = 2157). Data on complementary feeding practices and potential determinants were obtained by questionnaire at infant’s age of 6 months. Logistic regression models were used to investigate factors associated with early introduction of complementary feeding and infants’ consumption of non-recommended foods.

**Results:**

21.4% of infants had received complementary feeding before 4 months of age. At the age of 6 months, 20.2% of all infants were consuming sweet beverages daily and 16.5% were consuming snack foods daily. Younger maternal age, lower maternal educational level, absence or shorter duration of breastfeeding, parental conviction that “my child always wants to eat when he/she sees someone eating” and not attending day-care were independently associated with both early introduction of complementary feeding and the consumption of non-recommended foods. Higher maternal pre-pregnancy BMI and infant postnatal weight gain were associated only with early introduction of complementary feeding.

**Conclusions:**

We identified several demographical, biological, behavioral, psychosocial, and social factors associated with inappropriate complementary feeding practices. These findings are relevant for designing intervention programs aimed at educating parents.

**Trial registration:**

The trail is registered at Netherlands Trial Register, trail registration number: NTR1831. Retrospectively registered on May 29, 2009.

**Electronic supplementary material:**

The online version of this article (10.1186/s12889-019-6722-4) contains supplementary material, which is available to authorized users.

## Background

Complementary feeding for infants is defined as feeding solid foods and liquids other than breast milk or infant formula [1, 2]. Since 2001, the WHO has recommended that complementary feeding be introduced after the age of 6 months [2]. The European Society for Pediatric Gastroenterology, Hepatology and Nutrition (ESPGHAN) recommends introducing complementary feeding not before 17 weeks and no later than 26 weeks [1, 3]. In the Netherlands, *Jeugdgezondheidszorg* (preventive Youth Health Care) is a government-funded program for monitoring children’s health and development, and providing health promotion and disease prevention at set ages; the care is offered for free [4]. Approximately 95% of children in the Netherlands participate in this preventive Youth Health Care (henceforth YHC) program [4]. In line with ESPGHAN guidelines, the YHC guideline suggests introducing complementary feeding after the age of 4 months [5].

Despite the inconsistencies in the current guidelines regarding when to introduce complementary feeding, all guidelines agree that complementary feeding should not be introduced before the age of 4 months [1, 3, 5]. Although introducing complementary feeding earlier may contribute to more rapid weight gain during infancy [6–8] and increased risk of childhood obesity in affluent populations [9–12], the introduction of complementary feeding before 4 months is common in many countries. For instance, the percentage of infants introduced to complementary feeding before the age of 4 months was 37% in a birth cohort born in 2007 and 2008 in Northwest Italy [13], 30% across the UK in 2010 [14], and 40% among infants born between 2005 and 2007 participating in a national study in the US [15]. To the best of our knowledge, no study has reported the prevalence of introducing complementary feeding before 4 months in the Netherlands.

It is not only the timing of the introduction of complementary feeding that is important but also the type of food introduced. Current guidelines recommend avoiding foods high in fat, salt or sugar and low in nutritional value in the first year of life [3, 5, 16]. A high intake of foods such as sweet desserts [17] and sweet beverages [18] during infancy is associated with a high intake of these food types in later life, and with childhood overweight and obesity [17]. Furthermore, excessive intake of sugar-sweetened beverages during infancy may result in diarrhea [19], failure to thrive [20], tooth decay [21], and decline in the consumption of other nutritious foods. It has been shown that a substantial proportion of infants consume sweet beverages and snack foods such as chocolate, cookies, and chips [22–25].

To develop targeted interventions to discourage the early introduction of complementary feeding and the consumption of non-recommended food types among infants, it is important to identify the determinants of both practices. Previous studies have identified a range of maternal and infant related factors associated with early introduction of complementary feeding, such as maternal age, maternal educational level, and maternal Body Mass Index (BMI), infant size or postnatal weight gain, and the initiation and duration of breastfeeding [26–33]. Psychosocial factors have so far received scant research attention: one study has suggested that mothers may introduce complementary feeding earlier in response to the infant’s fussy temperament [34], another has shown the influence of certain parental beliefs about infant feeding and infant weight status [35]. Factors associated with infants’ consumption of non-recommended foods (i.e., energy-dense, nutrient-poor foods and sweet beverages) have also received little attention [24, 25]. Furthermore, the single study we found performed in the Netherlands on factors associated with the timing of complementary feeding introduction [29] did not assess factors associated with the introduction of complementary feeding before child age 4 months [29].

As complementary feeding practices have been found to differ between countries [36], in order to develop population-specific strategies it is important to explore factors associated with complementary feeding practices in different settings. Our study therefore aimed to investigate factors associated with inappropriate complementary feeding practices, including the early introduction of complementary feeding, and the consumption of non-recommended foods, including sweet beverages and snack foods, in a population-based sample of parents and children in the Netherlands. We considered a range of demographic, biological, behavioral, psychosocial, and social factors, and explored their associations with inappropriate complementary feeding practices.

## Methods

### Study design and study population

We performed secondary data analysis using data from the BeeBOFT study, which is a population-based cluster randomized controlled trial for the primary prevention of overweight among young children (0–3 years) in the Netherlands [37]. In total, 51 YHC teams covering urban and rural areas in the Netherlands participated. Each YHC organization serves a region of the Netherlands, and each YHC team within an organization serves one or more municipalities of the region [4]. A team comprises a physician, nurse, and assistant [4]. The 51 YHC teams were randomly allocated to three study arms, the “BBOFT+” intervention (17 teams), the “E-health4Uth” intervention (17 teams), or the control group (17 teams). At each routine YHC visit (scheduled at child ages of 0.5, 1, 2, 3, 4, 6, 9, 11, 14, 18, and 36 months), parents allocated to the “BBOFT+” group received an intervention on child-rearing skills concerning healthy behavioral lifestyle habits of the child from birth onward. Parents allocated to the “E-health4Uth” group received intervention twice: at child ages of circa 18 and 24 months. Parents in the control group received usual care. After reviewing the research proposal of the BeeBOFT study, the Erasmus University Medical Center Medical Ethics Committee concluded that the Dutch Medical Research Involving Human Subjects Act did not apply to it. The Medical Ethics Committee therefore had no objection to the execution of the BeeBOFT study (proposal number MEC-2008-250).

From January 2009 through September 2010, parents were invited to participate in the BeeBOFT study when one of the 51 participating YHC teams visited them at home 2–4 weeks after the birth of the child. In total, 3003 parents provided written informed consent and filled in the baseline questionnaire. At child age 6 months, all the parents were invited to complete a questionnaire regarding their child’s health-related behaviors, including timing of introduction of complementary feeding, the frequency of consumption of complementary feeding, and the determinants of these behaviors. A total of 2331 parents returned the questionnaire at child age 6 months (age range 6–8 months). The questionnaire asked about the timing of the introduction of 22 types of food. Children for whom values were missing for more than five food types were excluded (*n* = 48). We also excluded preterm babies (gestational age < 37 weeks, *n* = 126). Finally, 2157 parent-child dyads were included in the present study.

Compared with the 672 infants excluded due to non-response for the questionnaire, the infants whose parents have responded the questionnaire (*n* = 2331) at child age 6 months had higher educated (20.0% low educated VS 11.4% low educated, *p* < 0.01).

### Measurements

#### Infant complementary feeding

##### Timing of complementary feeding

At child age 6 months, parents were asked to report in the questionnaire at which age the child had received the following products (Additional file 1: Table S1): fruit juice; fruit juice concentrate; soft drinks (e.g., cola, iced tea); light soft drinks; fruit cordials or syrup; sweetened dairy drinks; milk or buttermilk; yogurt; porridge; bread; baby cookies; chocolate or candy; crackers or breadsticks; fruit from a jar; fresh fruit; vegetables from a jar; vegetables with fish or meat from a jar; pasta/rice/potato; fresh vegetable; fish/meat/meat substitutes. The response categories included: “< 1 month”, “between 1–2 months”, “between 2–3 months”, “between 3–4 months”, “between 4–5 months”, “older than 5 months”, and “never given”. Parents could choose “never given” if at the time they filled in the questionnaire they had not introduced that food item. For descriptive analysis, the response categories “< 1 month”, “between 1–2 months”, “between 2–3 months”, and “between 3–4 months” were combined into “before 4 months”. The average age of the infants when parents filled in the questionnaire was 6.3 months, SD = 0.6. The drinks fruit juice, fruit juice concentrate, soft drinks, fruit cordial or syrup, and sweetened dairy drinks were combined into one category called sweet beverages. The foods baby cookies and chocolate or candy were combined into one category called snack foods. The timing of introduction of complementary feeding was defined as the earliest time point that any of the abovementioned drinks and foods were first given to the child. Early introduction of complementary feeding was defined as introduction of complementary feeding (i.e., drinks and foods) before 4 months.

##### Frequent consumption of non-recommended foods

The questionnaire also assessed how frequently on average the child was given the abovementioned food products when parents filled in the questionnaire at 6 months (Additional file 1: Table S1). The response categories included: “never given”, “<once per week”, “1–3 times per week”, “4–6 times per week”, “1–2 times per day”, “3–4 times per day”, and “>5 times per day”. The non-recommended foods included sweet beverages and snack foods as defined above. Frequent consumption of non-recommended foods was defined as the consumption of sweet beverages and/or snack foods ≥1 time per day.

#### Independent variables

Based on previous research [26–32, 38], the following variables were selected as potential determinants for the early introduction of complementary feeding and consumption of non-recommended foods.

##### Demographic characteristics

The demographic characteristics obtained by the baseline questionnaire were maternal age (years), maternal educational level, maternal ethnic background (native/non-native), maternal employment status (employed/unemployed), family structure (single parent/two parents), child gender (girl/boy), parity (primipara/multipara), and gestational age (weeks). Maternal educational level was categorized as high (higher vocational training, university degree), middle (> 4 years general secondary school or intermediate vocational training), and low (no education, primary school, or 4 years or less general secondary school) [39]. The mother’s ethnic background was classified as non-native if one of her parents had been born outside the Netherlands [40].

##### Biological factors

Maternal pre-pregnancy weight and height were self-reported in the baseline questionnaire. Maternal pre-pregnancy BMI was calculated by weight (kg)/height^2^ (meters). Data on child weight at birth and at age 3 months were acquired from the YHC registration files. Child weight and height were measured by YHC professionals in accordance with standardized protocols at each routine visit (set at ages 0, 1, 2, 3, 4, 6 months) [41]. Child weight for age Z-score 7was calculated using the Dutch 1997 age- and gender- specific reference values [42]. Infant postnatal weight gain between age 0–3 months was calculated by subtracting the weight for age Z-score at birth from the weight for age Z-score at 3 months.

##### Behavioral factors

At child age 6 months, parents were asked to report whether they had started breastfeeding (yes, no), and, if so, how old the child was when the mother stopped breastfeeding (response categories included within 2 weeks, between 2 and 4 weeks, between 1 and 2 months, between 2 and 3 months, between 3 and 4 months, between 4 and 5 months, older than 5 months, and still breastfeeding) (Additional file 1: Table S1). The responses to these two questions led us to create a new variable indicating the duration of any breastfeeding: “no breastfeeding”, “breastfeeding for 0.5–4 months”, or “breastfeeding for 4 months or longer”.

##### Psychosocial factors

The psychosocial factors maternal depressive symptoms, parental beliefs, and infant temperament were assessed by parental questionnaire at child age 6 months. Maternal depressive symptoms were assessed using the 10-question Edinburgh Postnatal Depression Scale [43]. Mothers scoring 10 or higher were classified as having depressive symptoms. This variable was defined as missing if the questionnaire had been filled in by the father or another care giver (*n* = 107).

Parental beliefs/perceptions about infant characteristics, feeding, and infant weight were assessed. The items are based on a previous study investigating parental views on child overweight-related behaviors [44]. Example items included the following statements “My child always wants to eat when he/she sees someone eating”, “Fruit and vegetables can be given to the baby freely earlier than 4 months” and “I don’t like my child to be fat”. Parents could respond on a 5-point scale ranging from “strongly agree” to “strongly disagree”. The responses were dichotomized into “1” indicating agree/strongly agree, and “0” indicating neutral, disagree, or strongly disagree.

Infant temperament, e.g., soothability, distress to limitations, and distress to novel food, was measured using subscales from the Infant Behavior Questionnaire [45]. The subscales were chosen based on previous research on infant temperament and infant feeding [34]. An example item used to measure soothability was “When part of the child’s body was patted or stroked, how often did she/he calm down immediately?”, for distress to limitations, “When having to wait for food or liquids during the last week, how often did the child cry loudly”, and for distress to novel food, “When given a new food or liquid, how often did the child accept it immediately?”. Parents rated these specific child behaviors on a 7-point scale ranging from 1 (“Never”) to 7 (“always”).

##### Social factors

Day-care attendance of the infants was reported by parents in the questionnaire at child age 6 months. In addition, we included a variable entitled “intervention group” for the current study. Parents allocated to the “BBOFT+” study arm were defined as the “BBOFT+ intervention” group, while parents allocated to the control group or the “E-health” intervention group were combined to form a “no intervention” group.

### Statistical analysis

All statistical analyses were performed using SAS version 9.4. Descriptive statistics for the study population were presented in relation to the timing of the introduction of complementary feeding (< 4 months vs ≥4 months). Differences between the two groups were compared by independent sample *t* test for continuous variables, and by the *x*^2^ test for categorical variables.

Intra-class coefficients (ICC) for our outcome variables (early introduction of complementary feeding and consumption of non-recommended foods) were calculated to decide whether the outcome variables differed for the participating YHC teams. The ICCs for both outcome variables were 0.02, suggesting a very low intra-class correlation and therefore multilevel modeling was not used. In addition, we found no significant influence of the intervention group on both outcome variables (both *p* > 0.25). We therefore applied normal logistic regression analyses to the data on all available participants to assess the factors associated with the early introduction of complementary feeding and with the frequent consumption of non-recommended foods. First, univariate logistic regression models were fitted for each of the independent variables with the outcome variables. Second, independent variables that were significantly (*p* < 0.05) associated with the outcome variables in the univariate models were included in the multivariate model, to assess the independent association between the factors and outcome variables. The univariate and multivariate models were both adjusted for the exact age of the child.

## Results

### Sample characteristics

Table 1, which presents the characteristics of the mothers and infants in relation to the timing of the introduction of complementary feeding, shows that 11.3% of mothers were low educated, 36.0% were middle educated, and 52.7% high educated, and that 24.6% had not breastfed, whereas 40.6% of the mothers had breastfed but stopped doing so before the child was 4 months.

**Table 1:** Characteristics of the total study population (*n* = 2157)

Variable	Missing (N)	Age at introduction of complementary feeding		*p* value
		> 4 months N (%)	< 4 months N (%)	
Total		1695(78.58)	462 (21.42)	
Demographic characteristics				
Maternal age at child birth, years, mean (SD)	31	31.36(4.1)	29.7(4.3)	< 0.001
Maternal educational level	13			< 0.001
Low		147(8.7)	96(21.1)	
Middle		576(34.1)	195(42.8)	
High		967(57.2)	165(36.2)	
Maternal ethnic background, native	4	1533(90.3)	407(88.1)	0.15
Maternal employment status, employed	4	1449(85.6)	377(81.6)	0.04
Family structure, single parent	23	1659(98.7)	440(96.7)	< 0.01
Infant gender, boy	1	843(49.7)	263(57.1)	< 0.01
Parity, primipara	0	747(44.0)	251(54.3)	< 0.001
Biological factors				
Maternal pre-pregnancy BMI	5	24.0(4.3)	25.0(4.9)	0.02
Infant gestational age at birth, weeks, mean (SD)	0	39.8(1.0)	39.7(1.0)	0.10
Infant weight at birth, Z-score, mean (SD)	10	0.4(1.0)	0.3(1.0)	0.03
Infant postnatal weight gain, Z-score, mean (SD)	677	−0.7(0.8)	−0.5(0.9)	< 0.001
Behavioral factors				
Duration of any breastfeeding	6			< 0.001
No breastfeeding		360(21.3)	169(36.8)	
Breastfeeding for 0.5–4 months		547(19.4)	207(26.8)	
Breastfeeding for 4 months or longer		789(46.6)	83(18.1)	
Psychosocial factors				
Maternal depressive symptom, yes^a^	119	1445(89.7)	371(86.5)	0.054
*Parental perceptions on infant characteristics, (agree/strongly agree)*				
“My baby drinks greedily”	23	477(28.4)	135(29.6)	0.07
“My child always wants to eat when he/she sees someone eating”	23	559(33.3)	212(46.6)	< 0.001
“My child does not like plain water”	45	293(17.6)	114(25.2)	< 0.001
“My child cried a lot in the first 3 months”	16	300(17.8)	99(21.6)	0.06
*Parental beliefs about feeding, (agree/strongly agree)*				
“Fruit and vegetables can be given to the baby freely earlier than 4 months”	20	39(2.3)	70(15.4)	< 0.001
*Parental beliefs about infant weight, (agree/strongly agree)*				
“I don’t like my child to be fat”	20	1206(71.7)	296(64.9)	< 0.01
“I don’t like my child to be thin”	15	924(54.8)	234(51.1)	0.18
*Infant temperament*				
Soothability, mean (SD)	31	4.8(1.2)	4.7(1.3)	0.06
Distress to limitation, mean (SD)	33	2.8(0.9)	2.7(0.9)	0.27
Distress to novel food, mean (SD)	36	2.3(1.4)	2.2(1.3)	0.02
Social care factors				
Day-care attendance, yes	25	1250(74.4)	297(65.4)	< 0.001
“BBOFT+” intervention^b^	0	509(30.0)	127(27.5)	0.27

^a^Maternal depressive symptom was defined as a score of 10 or greater on the Edinburgh Postnatal Depression Scale. This variable was defined as missing if the questionnaire had been filled in by the father or other care givers (*n* = 107)

^b^The “BBOFT+ Intervention” group comprised the group of parents allocated to the BBOFT+ study arm; “no intervention” comprised the groups of parents allocated to the control group or to the “E-health” intervention group

### Complementary feeding practices

Table 2 presents the timing of the introduction of different types of complementary food. Overall, the percentage of infants who had been given any type of complementary food at the age of 3, 4, and 5 months was 4.5% (data not shown in table), 21.4, and 38.1% respectively. At the moment parents filled in the questionnaire (mean age = 6.4 months, SD = 0.7), 98.7% of the infants had been given some type of complementary food. The food products most frequently introduced before 4 months were porridge (11.8%), fruit (11.0%), vegetables (6.4%), and sweet beverages (6.1%).

**Table 2:** The timing of introduction of different types of complementary food (*N* = 2157)

Type of complementary food	Before 4 months	Between 4–5 months	After age 5 months^a^	Never given^b^
	N (%)	N (%)	N (%)	N (%)
Sweet beverages^c^	132(6.1)	251(11.6)	740(34.3)	1036(48.0)
Milk or buttermilk	18(0.8)	8(0.4)	57(2.7)	2067(96.1)
Yogurt	32(1.5)	99(4.6)	611(28.4)	1413(65.6)
Porridge	255(11.8)	605(28.0)	719(33.3)	580(26.9)
Bread	10(0.5)	81(3.8)	1019(47.2)	1047(48.5)
Snack foods^d^	16(0.7)	124(5.7)	713(33.0)	1306(60.5)
Crackers or breadsticks	4(0.2)	55(2.6)	484(22.5)	1610(74.8)
Fruit	236(11.0)	791(36.7)	1067(49.5)	62(2.9)
Vegetables	137(6.4)	638(29.6)	1240(57.4)	144(6.7)
Pasta/potato/rice	16(0.7)	112(5.2)	1072(49.8)	952(44.2)
Fish/meat/meat substitutes	34(1.6)	163(7.6)	1120(51.9)	841(39.0)
Any complementary food	462(21.4)	875(40.5)	794(36.8)	28(1.3)

^a^After the child reached the age of 5 months, and before the time parent completed the questionnaire on infant feeding. The mean age of the infants at questionnaire completion was 6.3 months (SD = 0.6)

^b^Complementary feeding had not yet been introduced to the infant when parents filled in the questionnaire

^c^Including fruit juice, fruit juice concentrate, soft drinks (e.g. cola, iced tea), fruit cordials or syrup, and sweetened dairy products

^d^Including baby cookies, and chocolate or candy

**Fig. 1:**
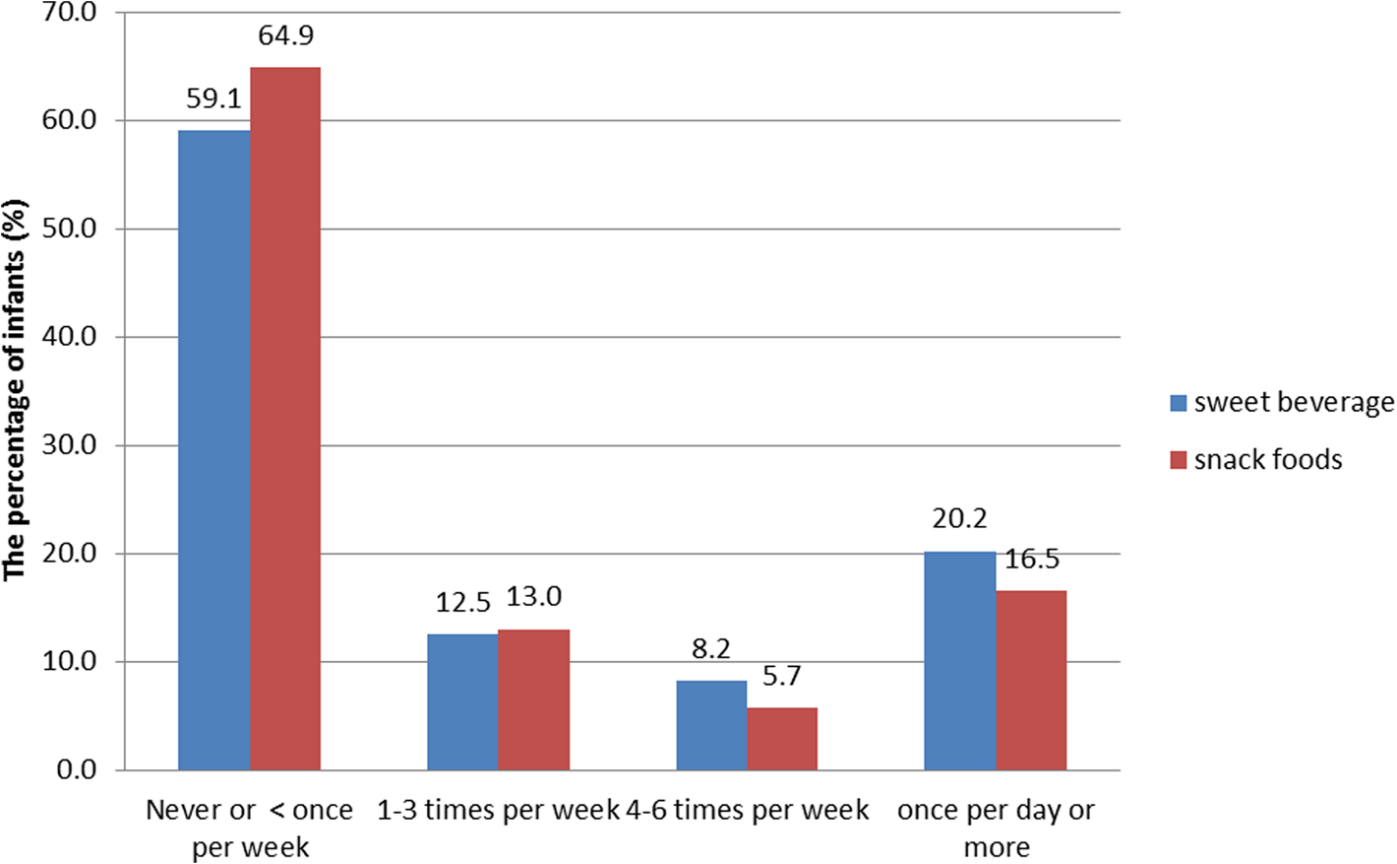
The frequency of consumption of non-recommended foods by the infant at the age of 6 months (*n* = 2157)

Figure 1 presents the frequency of consumption of sweet beverages and snack foods by the infants. At the age of 6 months, 41% of the infants were consuming sweet beverages at least once a week and 20.2% of the infants were consuming sweet beverages daily. In addition, 35% of the infants were consuming snack food at least once weekly, and 16.5% of the infants were consuming snack food daily. In total, 27.0% of the infants were consuming non-recommended foods (i.e., sweet beverages and/or snack food) at least once daily.

### Factors associated with early introduction of complementary feeding

Table 3 shows the results of univariate and multivariate logistic regression models for factors associated with early introduction of complementary feeding (i.e., introduction of complementary feeding before child age 4 months). The demographic characteristics independently associated with early introduction of complementary feeding were younger maternal age and lower maternal educational level. For biological factors: increased maternal pre-pregnancy BMI and increased infant postnatal weight gain were independently associated with higher odds of early introduction of complementary feeding. For behavioral factors: compared to any breastfeeding for 4 months or longer, no breastfeeding or breastfeeding for less than 4 months was independently associated with early introduction of complementary feeding. For psychosocial factors: the beliefs “fruit and vegetables can be given to the baby freely earlier than 4 months”, and “my child always wants to eat when he/she sees someone eating” were independently associated with higher odds of early introduction of complementary feeding. For social factors, day-care attendance was independently associated with lower odds of early introduction of complementary feeding.

**Table 3:** Factors associated with early introduction of complementary feeding

	Early introduction of complementary feeding < 4 months vs > 4 months	
	Univariate models	Multivariate model
	OR (95%CI)	OR (95%CI)
Demographic characteristics		
Maternal age at child birth (years)	0.91(0.88,0.93)^***^	0.94(0.91,0.98)^**^
Maternal educational level		
Low vs high	3.82(2.82,5.19)^***^	2.48(1.57,3.92)^***^
Middle vs high	1.98(1.57,2.50)	1.26(0.91,1.75)
Maternal ethnic background, non-native vs native	1.23(0.88,1.71)	
Maternal employment status, unemployed vs employed	1.33(1.02,1.75)^*^	0.90(0.57,1.39)
Family structure, single parent vs two parents	2.56(1.32,4.97)^**^	1.88(0.69,5.13)
Infant gender, girl vs boy	0.74(0.60,0.92)^**^	0.90(0.67,1.20)
Parity, multipara vs primipara	0.66(0.54,0.81)^***^	0.79(0.58,1.08)
Biological factors		
Maternal pre-pregnancy BMI	1.05(1.02,1.07)^***^	1.02(1.00,1.06)
Infant gestational age (weeks)	0.92(0.83,1.02)	
Infant weight at birth, Z-score	0.82(0.66,1.01)	
Infant postnatal weight gain, Z-score^a^	1.33(1.15,1.55)^***^	1.24(1.05,1.50)^*^
Behavioral factor		
Duration of any breastfeeding		
No breastfeeding	4.47(3.34,5.97)^***^	2.84(1.90,4.30)^*^
Breastfeeding for 0.5–4 months	3.62(2.75,4.78)^**^	2.63(1.82,3.80)^*^
Breastfeeding for 4 months or longer	Ref	Ref
Psychosocial factors		
Maternal depressive symptoms, yes vs no ^b^	1.35(0.98,1.86)	
*Parental perceptions on infant characteristics* ^c^		
“My child drinks greedily”	1.06(0.85,1.34)	
“My child always wants to eat when he/she sees someone eating”	1.75(1.42,2.16)^***^	1.50(1.11,2.01)^**^
“My child does not like plain water”	1.58(1.23,2.02)^***^	1.08(0.76,1.54)
“My child cried a lot in the first 3 months”	1.28(0.99,1.65)	
*Parental belief about feeding* ^c^		
“Fruit and vegetables can be given to the baby freely earlier than 4 months”	7.61(5.07,11.44)^***^	5.60(3.18,9.85)^***^
*Parental beliefs about infant weight* ^c^		
“I don’t like my child to be fat”	0.73(0.59,0.91)^**^	0.82(0.59,1.11)
“I don’t like my child to be thin”	0.86(0.70,1.06)	
*Infant temperament*		
Soothability	0.93(0.86,1.01)	
Distress to limitations	0.94(0.83,1.05)	
Distress to novel food	0.91(0.84,0.98)	0.90(0.80,1.01)
Social care factors		
Day-care attendance, yes vs no	0.65(0.52,0.81)^**^	0.66(0.47,0.93)^*^
“BBOFT+” intervention vs no intervention	0.89(0.70,1.11)	

Note: The multivariate model included the factors significantly (*p* < 0.05) associated with the outcome variable in the univariate models

^*^p < 0.05, ^**^p < 0.01, ^***^p < 0.001, *p* < 0.10

^a^Calculated by changes in weight for age Z-scores in the first 3 months

^b^Maternal depressive symptom was defined as scored 10 or greater on the Edinburgh Postnatal Depression Scale. This variable was defined as missing if the questionnaire had been filled in by the father or other care givers (*n* = 107)

^c^Agree/strongly agree vs neutral, disagree, or strongly disagree

### Factors associated with frequent consumption of non-recommended foods

The results of the multivariate logistic regression model (Table 4) suggest that younger maternal age, lower maternal educational level, and no breastfeeding or breastfeeding for less than 4 months were associated with frequent consumption of non-recommended foods (once or more per day) of the infants at the age of 6 months. Of the psychosocial factors, the beliefs “fruit and vegetables can be given to the child freely earlier than 4 months” and “my child always wants to eat when he/she sees someone eating” were associated with frequent consumption of non-recommended foods. Infant temperament “soothability” was positively associated with frequent consumption of non-recommended foods, while “distress to novel food” was negatively associated with frequent consumption of non-recommended foods. Of the social factors, day-care attendance was associated with lower consumption of non-recommended foods.

**Table 4:** Factors associated with the consumption of non-recommended foods

	Frequent consumption of non-recommended foods ≥ once per day vs < once per day	
	Univariate models	Multivariate model
	OR (95%CI)	OR (95%CI)
Demographic characteristics		
Maternal age at child birth (years)	0.93(0.91,0.96)^***^	0.96(0.94,0.99)^*^
Maternal educational level		
Low vs high	2.90(2.15,3.92)^***^	2.02(1.42,2.86)^***^
Middle vs high	1.73(1.40,2.15)^*^	1.36(1.07,1.73)^*^
Maternal ethnic background, non-native vs native	1.28(0.94,1.75)	
Maternal employment status, unemployed vs employed	1.23(0.95,1.60)	
Family structure, single parent vs two parents	1.36(0.67,2.76)	
Infant gender, girl vs boy	0.79(0.65,0.97)^*^	0.86(0.70,1.06)
Parity, multipara vs primipara	0.77(0.63,0.93)^**^	0.85(0.68,1.08)
Biological factors		
Maternal pre-pregnancy BMI	1.01(0.99,1.03)	
Infant gestational age (weeks)	0.90(0.82,1.00)^*^	0.94(0.84,1.04)
Infant weight at birth, Z-score	0.94(0.84,1.04)	
Infant postnatal weight gain, Z-score^a^	0.92(0.80,1.06)	
Behavioral factor		
Duration of any breastfeeding		
No breastfeeding	2.37(1.85,3.03)^***^	1.91(1.44,2.52)^***^
Breastfeeding for 0.5–4 months	1.51(1.19,1.91)^*^	1.35(1.04,1.74)^*^
Breastfeeding for 4 months or longer	Ref	Ref
Psychosocial factors		
Maternal depressive symptoms, yes vs no^b^	1.14(0.83,1.55)	
*Parental perceptions on characteristics* ^c^		
“My child drinks greedily”	0.81(0.65,1.01)	
“My child always wants to eat when he/she sees someone eating”	1.59(1.30,1.94)^***^	1.44(1.16,1.79)^***^
“My child does not like plain water”	1.31(1.03,1.67)^*^	1.08(0.83,1.41)
“My child cried a lot in the first 3 months”	0.93(0.72,1.20)	
*Parental belief about feeding* ^c^		
“Fruit and vegetables can be given to the baby freely earlier than 4 months”	2.36(1.58,3.52)^*^	1.66(1.07,2.56)^*^
*Parental beliefs about infant weight* ^c^		
“I don’t like my child to be fat”	0.78(0.63,0.96)^*^	0.80(0.64,1.01)
“I don’t like my child to be thin”	0.96(0.79,1.17)	
*Infant temperament*		
Soothability	1.12(1.04,1.22)^***^	1.15(1.06,1.26)^**^
Distress to limitations	1.00(0.90,1.12)	
Distress to novel food	0.91(0.85,0.98)^*^	0.92(0.85,0.99)^*^
Social care factors		
Day-care attendance, yes vs no	0.63(0.51,0.78)^***^	0.76(0.60,0.96)^*^
“BBOFT+” intervention vs no intervention	0.91(0.73,1.13)	

Note: Both the univariate models and the multivariate model adjusted for age at questionnaire measurement. The multivariate model included the factors significantly (*p* < 0.05) associated with the outcome variable in the univariate models

^a^Calculated by changes in weight for age Z-scores in the first 3 months

^b^Maternal depression symptom was defined as scored 10 or greater on the Edinburgh Postnatal Depression Scale. This variable was defined as missing if the questionnaire had been filled in by the father or other care givers (*n* = 107)

^c^Agree/strongly agree vs neutral, disagree, or strongly disagree

^*^p < 0.05, ^**^p < 0.01, ^***^p < 0.001,

## Discussion

In our population-based sample of parent–child dyads from the Netherlands, 21% of the infants were introduced to complementary feeding before the age of 4 months, and 38% of the infants were introduced to complementary feeding after 5 months. Less than 2% of the infants had not received any complementary feeding at the moment of questionnaire completion (mean age of the infants then was 6.3 months). In addition, we observed that a significant proportion of the infants were consuming sweet beverages or snack food at age 6 months, pointing to a need to put greater emphasis on discouraging giving sweet beverages and snack foods to infants.

In line with previous research [25, 26, 33, 46], we found that mothers who were younger, less educated, and who did not initiate breastfeeding or breastfed for shorter duration were more likely to introduce complementary feeding early. In addition, our results suggest that these factors are also associated with frequent consumption of non-recommended foods among the infants. Our findings underline the need to develop effective interventions targeting these groups of mothers (i.e., younger, lower educated, and not breastfeeding) to improve their feeding practices, including the timing and types of complementary feeding.

Our results suggest that mothers with higher pre-pregnancy BMI were more likely to introduce complementary feeding early. Previous studies have suggested that maternal pre-pregnancy overweight/obesity is linked to impaired lactogenesis [47–49]. Overweight or obese mothers may have difficulty initiating or sustaining breastfeeding, and therefore may introduce complementary feeding earlier to compensate for the insufficiencies in breastmilk. In line with this hypothesis, we found that the association between maternal pre-pregnancy BMI and early introduction of complementary feeding was reduced to borderline significance after adjusting for breastfeeding duration (data not shown).

Our results reveal that infants with more rapid postnatal weight gain were more likely to receive complementary feeding early. This finding is consistent with previous evidence [6, 50, 51]. Our study further confirmed that rapid postnatal weight gain was associated with early introduction of complementary feeding independent of factors such as breastfeeding duration. A possible explanation for this association is that infants who grow faster in the first few months may show more hunger cues, or signs of readiness for complementary feeding. Rapid weight gain in the first few months is associated with increased risk of overweight [52–54], and cardiovascular risk factors in later life [55–57]. Early introduction of complementary feeding may further increase infants’ energy intake and growth velocity [6–8]. Future research investigating the influence of complementary feeding practices on infant weight gain should be aware of the reverse causality: that rapid postnatal weight gain may induce early introduction of complementary feeding.

Our study further revealed that psychosocial factors play an important role in parents’ adoption of complementary feeding practices. We identified several parental perceptions/beliefs concerning infant characteristics and infant weight that may contribute to inappropriate complementary feeding practices. For instance, parents who perceived that “my child always wants to eat when he/she sees someone eating” and parents who agree with the idea that “fruit and vegetables can be given to the child freely earlier than 4 months” were more likely to introduce complementary feeding early, and to give their infants non-recommended foods more frequently. We are aware of only one study that has included the parental perceptions or beliefs as determinants of infant complementary feeding [58], and comparison with that study is difficult because the outcome variables was defined differently. As psychosocial factors tend to be more modifiable than demographical and biological factors, in future intervention programs it would be beneficial to target these psychosocial contributors for inappropriate complementary feeding. In view of the cross-sectional nature of our data, no causal relationship can be inferred from the present study. We recommend further longitudinal studies or controlled trials to confirm our findings. In addition, we recommend conducting further qualitative or quantitative to obtain more thorough understanding of the psychosocial factors contributing to inappropriate complementary feeding.

With regard to social factors, we found that infants who attended day-care were less likely to receive complementary feeding early and were less likely to consume non-recommended foods frequently. Previous studies conducted in other countries have found no association between day-care attendance and early introduction of complementary feeding [33]. However, differences in the overall child-care systems in different countries (for example, different policies, social norms), might have influenced the findings. Consistent with our study, a previous study in the Netherlands suggested that day-care attendance is associated with less unhealthy lifestyles of young children [44]. It has also been reported that day-care attendance in the first year of life was associated with better general health and lower risk of overweight and obesity of the children across the age span of 1 to 8 years in a birth cohort from the Netherlands [59]. The association of day-care use and more favorable infant feeding practices in the present study and more favorable lifestyles and general health of children found in previous studies might reflect other characteristics of families using day-care facilities. In our study, the mothers of children who attended day-care at age 6 months were more often higher educated, employed, and less often overweight. We recommend further studies to investigate the reasons for the role of day-care attendance on children’s healthy lifestyles and health outcomes.

A limitation of the present study is that the timing of introduction of complementary feeding was self-reported by parents retrospectively. However, the data were collected when infants were 6 months, which was close to the time of introduction of complementary feeding. This may have reduced the recall bias on timing of introduction of complementary feeding. Secondly, it should be noted that the participants who responded to the questionnaire had a higher educational level and higher rate of breastfeeding than those who did not. Our study may therefore have underestimated the proportion of infants in the population who had received complementary feeding before 4 months. Thirdly, it is a limitation of the present study that we were unable to precisely estimate the percentage of infants who were introduced to complementary feeding after the age of 6 months. However, this study followed the ESPHAGAN recommendation adopted by many countries in Europe, which defines early introduction of complementary feeding as the introduction of complementary feeding before 4 months [3, 5, 36]. Finally, our study used data from a cluster randomized controlled trial for prevention of childhood overweight [37]. Parents allocated to the BBOFT+ group received intervention on child-rearing practices from birth onwards. The intervention did not include specific information on timing of the introduction of complementary feeding. The intervention is unlikely to have influenced our results, as the ICC was low and a sensitivity analysis using a sample from the control group generated comparable results.

## Conclusions

In conclusion, the present study addresses the need to improve the compliance with complementary feeding guidelines among parents in the Netherlands, more specifically the introduction of complementary feeding after age 4 months, and the avoidance of giving infants sweet beverages and snack foods. Factors associated with inappropriate complementary feeding practices include younger maternal age, lower maternal educational level, absence or shorter duration of breastfeeding, increased maternal pre-pregnancy BMI and infant postnatal weight gain, and not attending day-care. We also identified several psychosocial factors associated with inappropriate complementary feeding practices. These findings are relevant for designing targeted interventions aimed at educating parents to improve their complementary feeding practices.

## Additional file

Additional file 1: **Table S1.** Questionnaires on infant feeding used in the present study. (DOCX 25 kb)

## Abbreviations

BMI: Body Mass Index
ESPGHAN: European Society for Pediatric Gastroenterology, Hepatology and Nutrition
ICC: Intra-class coefficient
SD: Standard Deviation
YHC: Youth Health Care
