# Supplementary material for: Factors associated with early introduction of complementary feeding and consumption of non-recommended foods among Dutch infants: the BeeBOFT study
Source: BMC Public Health. 2019 Apr 8;19:388. doi: 10.1186/s12889-019-6722-4 (PMC6454678; doi:10.1186/s12889-019-6722-4)
Supplement: Supplementary file 1 — Table S1. Questionnaires on infant feeding used in the present study. (DOCX 25 kb) [file 12889_2019_6722_MOESM1_ESM.docx]

Additional file 1: Table S1. Questionnaires on infant feeding used in the present study

| **Infant feeding** | |
| --- | --- |
| 1. Please indicate how old was your child when you gave the following products for the first time ? | |
| Fruit Juice | 1) <1 month, 2) between 1–2 months, 3) between 2–3 months, 4) between 3–4 months, 5) between 4–5 months, 6) older than 5 months, 7) never given; |
| Fruit juice concentrate | 1) <1 month, 2) between 1–2 months, 3) between 2–3 months, 4) between 3–4 months, 5) between 4–5 months, 6) older than 5 months, 7) never given; |
| Sweetened beverages/ soft drinks (eg. Cola, ice-tea) | 1) <1 month, 2) between 1–2 months, 3) between 2–3 months, 4) between 3–4 months, 5) between 4–5 months, 6) older than 5 months, 7) never given; |
| Light soft drinks | 1) <1 month, 2) between 1–2 months, 3) between 2–3 months, 4) between 3–4 months, 5) between 4–5 months, 6) older than 5 months, 7) never given; |
| Fruit cordials or syrup | 1) <1 month, 2) between 1–2 months, 3) between 2–3 months, 4) between 3–4 months, 5) between 4–5 months, 6) older than 5 months, 7) never given; |
| Sweetened dairy drinks | 1) <1 month, 2) between 1–2 months, 3) between 2–3 months, 4) between 3–4 months, 5) between 4–5 months, 6) older than 5 months, 7) never given; |
| Milk or buttermilk | 1) <1 month, 2) between 1–2 months, 3) between 2–3 months, 4) between 3–4 months, 5) between 4–5 months, 6) older than 5 months, 7) never given; |
| Yogurt | 1) <1 month, 2) between 1–2 months, 3) between 2–3 months, 4) between 3–4 months, 5) between 4–5 months, 6) older than 5 months, 7) never given; |
| Porridge in a bottle | 1) <1 month, 2) between 1–2 months, 3) between 2–3 months, 4) between 3–4 months, 5) between 4–5 months, 6) older than 5 months, 7) never given; |
| Porridge from a plate | 1) <1 month, 2) between 1–2 months, 3) between 2–3 months, 4) between 3–4 months, 5) between 4–5 months, 6) older than 5 months, 7) never given; |
| Bread | 1) <1 month, 2) between 1–2 months, 3) between 2–3 months, 4) between 3–4 months, 5) between 4–5 months, 6) older than 5 months, 7) never given; |
| Baby cookies | 1) <1 month, 2) between 1–2 months, 3) between 2–3 months, 4) between 3–4 months, 5) between 4–5 months, 6) older than 5 months, 7) never given; |
| Chocolate or candy | 1) <1 month, 2) between 1–2 months, 3) between 2–3 months, 4) between 3–4 months, 5) between 4–5 months, 6) older than 5 months, 7) never given; |
| Crackers, grissini | 1) <1 month, 2) between 1–2 months, 3) between 2–3 months, 4) between 3–4 months, 5) between 4–5 months, 6) older than 5 months, 7) never given; |
| Fruit from jars | 1) <1 month, 2) between 1–2 months, 3) between 2–3 months, 4) between 3–4 months, 5) between 4–5 months, 6) older than 5 months, 7) never given; |
| Fresh fruit | 1) <1 month, 2) between 1–2 months, 3) between 2–3 months, 4) between 3–4 months, 5) between 4–5 months, 6) older than 5 months, 7) never given; |
| Vegetable from a jar without meat or fish | 1) <1 month, 2) between 1–2 months, 3) between 2–3 months, 4) between 3–4 months, 5) between 4–5 months, 6) older than 5 months, 7) never given; |
| Vegetable from a jar with meat or fish | 1) <1 month, 2) between 1–2 months, 3) between 2–3 months, 4) between 3–4 months, 5) between 4–5 months, 6) older than 5 months, 7) never given; |
| Potatoes/rice/pasta | 1) <1 month, 2) between 1–2 months, 3) between 2–3 months, 4) between 3–4 months, 5) between 4–5 months, 6) older than 5 months, 7) never given; |
| Fresh vegetable | 1) <1 month, 2) between 1–2 months, 3) between 2–3 months, 4) between 3–4 months, 5) between 4–5 months, 6) older than 5 months, 7) never given; |
| Meat/fish/chicken/ meat substitutes | 1) <1 month, 2) between 1–2 months, 3) between 2–3 months, 4) between 3–4 months, 5) between 4–5 months, 6) older than 5 months, 7) never given; |
| 2. How often on average do you give your child the following food products at this moment? | |
| Fruit Juice (50-100 ml) | 1) never given, 2) <once per week, 3) 1–3 times per week, 4) 4–6 times per week, 5) 1–2 times per day, 6) 3–4 times per day, 7) >5 times per day; |
| Fruit juice concentrate (50-100 ml) | 1) never given, 2) <once per week, 3) 1–3 times per week, 4) 4–6 times per week, 5) 1–2 times per day, 6) 3–4 times per day, 7) >5 times per day; |
| Sweetened beverages / soft drinks (eg. Cola, ice-tea) (50-100 ml) | 1) never given, 2) <once per week, 3) 1–3 times per week, 4) 4–6 times per week, 5) 1–2 times per day, 6) 3–4 times per day, 7) >5 times per day; |
| Light soft drinks (50-100 ml) | 1) never given, 2) <once per week, 3) 1–3 times per week, 4) 4–6 times per week, 5) 1–2 times per day, 6) 3–4 times per day, 7) >5 times per day; |
| Fruit cordials or syrup (50-100 ml) | 1) never given, 2) <once per week, 3) 1–3 times per week, 4) 4–6 times per week, 5) 1–2 times per day, 6) 3–4 times per day, 7) >5 times per day; |
| Sweetened dairy drinks (50-100 ml) | 1) never given, 2) <once per week, 3) 1–3 times per week, 4) 4–6 times per week, 5) 1–2 times per day, 6) 3–4 times per day, 7) >5 times per day; |
| Milk or buttermilk (50-100 ml) | 1) never given, 2) <once per week, 3) 1–3 times per week, 4) 4–6 times per week, 5) 1–2 times per day, 6) 3–4 times per day, 7) >5 times per day; |
| Yogurt | 1) never given, 2) <once per week, 3) 1–3 times per week, 4) 4–6 times per week, 5) 1–2 times per day, 6) 3–4 times per day, 7) >5 times per day; |
| Porridge in a bottle | 1) never given, 2) <once per week, 3) 1–3 times per week, 4) 4–6 times per week, 5) 1–2 times per day, 6) 3–4 times per day, 7) >5 times per day; |
| Porridge from a plate | 1) never given, 2) <once per week, 3) 1–3 times per week, 4) 4–6 times per week, 5) 1–2 times per day, 6) 3–4 times per day, 7) >5 times per day; |
| Bread | 1) never given, 2) <once per week, 3) 1–3 times per week, 4) 4–6 times per week, 5) 1–2 times per day, 6) 3–4 times per day, 7) >5 times per day; |
| Baby cookies (1 piece) | 1) never given, 2) <once per week, 3) 1–3 times per week, 4) 4–6 times per week, 5) 1–2 times per day, 6) 3–4 times per day, 7) >5 times per day; |
| Chocolate or candy (1 piece) | 1) never given, 2) <once per week, 3) 1–3 times per week, 4) 4–6 times per week, 5) 1–2 times per day, 6) 3–4 times per day, 7) >5 times per day; |
| Crackers, grissini (1 piece) | 1) never given, 2) <once per week, 3) 1–3 times per week, 4) 4–6 times per week, 5) 1–2 times per day, 6) 3–4 times per day, 7) >5 times per day; |
| Fruit from jars | 1) never given, 2) <once per week, 3) 1–3 times per week, 4) 4–6 times per week, 5) 1–2 times per day, 6) 3–4 times per day, 7) >5 times per day; |
| Fresh fruit | 1) never given, 2) <once per week, 3) 1–3 times per week, 4) 4–6 times per week, 5) 1–2 times per day, 6) 3–4 times per day, 7) >5 times per day; |
| Vegetable from a jar without meat or fish | 1) never given, 2) <once per week, 3) 1–3 times per week, 4) 4–6 times per week, 5) 1–2 times per day, 6) 3–4 times per day, 7) >5 times per day; |
| Vegetable from a jar with meat or fish | 1) never given, 2) <once per week, 3) 1–3 times per week, 4) 4–6 times per week, 5) 1–2 times per day, 6) 3–4 times per day, 7) >5 times per day; |
| Potatoes/rice/pasta | 1) never given, 2) <once per week, 3) 1–3 times per week, 4) 4–6 times per week, 5) 1–2 times per day, 6) 3–4 times per day, 7) >5 times per day; |
| Fresh vegetable | 1) never given, 2) <once per week, 3) 1–3 times per week, 4) 4–6 times per week, 5) 1–2 times per day, 6) 3–4 times per day, 7) >5 times per day; |
| Meat/fish/chicken/ meat substitutes | 1) never given, 2) <once per week, 3) 1–3 times per week, 4) 4–6 times per week, 5) 1–2 times per day, 6) 3–4 times per day, 7) >5 times per day; |
| 3. Whether the child have been breastfed? | |
| 1) Yes, 2) No | |
| 4. How old the child was when the mother stopped breastfeeding | |
| 1) I am still giving breastfeeding  2) within 2 weeks,  3) between 2 and 4 weeks,  4) between 1 and 2 months, between 2 and 3 months  5) between 3 and 4 months  6) between 4 and 5 months, older than 5 months | |
